# Photoinduced bidirectional mesophase transition in vesicles containing azo­benzene amphiphiles

**DOI:** 10.1107/S2052252524004032

**Published:** 2024-05-28

**Authors:** Svenja C. Hövelmann, Ella Dieball, Jule Kuhn, Michelle Dargasz, Rajendra P. Giri, Franziska Reise, Michael Paulus, Thisbe K. Lindhorst, Bridget M. Murphy

**Affiliations:** a Institute of Experimental and Applied Physics Kiel University Leibnizstraße 19 24118 Kiel Germany; b Deutsche Elektronen-Synchrotron DESY Notkestraße 85 22607 Hamburg Germany; c Ruprecht Haensel Laboratory, Olshausenstraße 40, 24098 Kiel, Germany; d Department Physik Universität Siegen Walter-Flex-Strasse 3 57072 Siegen Germany; e Otto Diels Institute of Organic Chemistry Kiel University Otto-Hahn-Platz 3-4 24118 Kiel Germany; f Fakultät Physik/DELTA, TU Dortmund, 44221 Dortmund, Germany; Uppsala University, Sweden

**Keywords:** lipid mesophase, photoswitches, azo­benzene, vesicles, small-angle X-ray scattering, light-induced mesophase transformations, temperature-induced mesophase changes, structure determination, solution scattering, structural biology, SAXS

## Abstract

The functionality and efficiency of proteins within a biological membrane are highly dependent on both the membrane lipid composition and the physiochemical properties of the solution. Lipid mesophases are directly influenced by changes in temperature, pH, water content or due to individual properties of single lipids such as photoswitchability. In this work, we were able to induce light- and temperature-driven mesophase transitions in a model membrane system containing a mixture of 1,2-dipalmitoyl-phosphatidylcholine phospho­lipids and azo­benzene amphiphiles. We observed reversible and reproducible transitions between the lamellar and *Pn*3*m* cubic phase after illuminating the sample for 5 min with light of 365 and 455 nm wavelengths, respectively, to switch between the *cis* and *trans* states of the azo­benzene N=N double bond. These light-controlled mesophase transitions were found for mixed complexes with up to 20% content of the photosensitive molecule and at temperatures below the gel-to-liquid crystalline phase transition temperature of 33°C. Our results demonstrate the potential to design bespoke model systems to study the response of membrane lipids and proteins upon changes in mesophase without altering the environment and thus provide a possible basis for drug delivery systems.

## Abstract 2

In this work, a reversible non-invasive light-induced mesophase transition from lamellar to cubic *Pn*3*m* is observed in mixed azo­benzene-lipid and phospho­lipid vesicles using small-angle X-ray scattering.

## 1. Introduction

Lipid membranes are a fundamental part of biological cells and contain various components such as phospho­lipids, cholesterol and proteins. Phospho­lipids are the main component of bilayer membranes and their composition determines the membrane properties and mesophase (Shah *et al.*, 2001 ▸; Yang *et al.*, 2012 ▸; Cavalcanti *et al.*, 2006 ▸; van Meer *et al.*, 2008 ▸). The lipid mesophase is of great interest for efficient drug transportation and release of pharmaceuticals because of the possibility of opening the water channel via phase changes, or due to its influence on membrane protein activity and efficiency (Shah *et al.*, 2001 ▸; Sagalowicz *et al.*, 2006 ▸; Cournia *et al.*, 2015 ▸; Hirlekar *et al.*, 2010 ▸). Lipid mesophases have multiple geometric shapes and structures which evolve when amphiphilic lipids such as phospho­lipids come in contact with water. The explicit geometric shape depends on multiple properties such as temperature, pH, salinity, volume concentration (% *w*/*w*) and lipid curvature, which can be quantified by the critical packing parameter *CPP* (McManus *et al.*, 2003 ▸; Di Cola *et al.*, 2016 ▸; Shah *et al.*, 2001 ▸). Changes in these properties can lead to structural changes and even a transition into another mesophase. These transitions can be used in bio-hybrid microsystems as biological actuators and sensors (Carlsen & Sitti, 2014 ▸). Different approaches to induce phase transitions have been studied extensively for application in drug delivery systems or for manipulation of proteins. Previous approaches employ temperature and water content (% *w*/*w*) (Shah *et al.*, 2001 ▸; Kulkarni, 2012 ▸), pH (Ribeiro *et al.*, 2019 ▸) or salinity (Muir *et al.*, 2012 ▸; Kalvodova *et al.*, 2005 ▸) to induce a mesophase transition. Light-induced structural changes (Basílio & García-Río, 2017 ▸) are mostly studied on lipid monolayers (Backus *et al.*, 2011 ▸; Warias *et al.*, 2023 ▸) and bilayers at the water–gas interface, or on small unilamellar vesicles (Urban *et al.*, 2020 ▸; Oh *et al.*, 2013 ▸; Ober *et al.*, 2022 ▸) and giant unilamellar vesicles (Pfeffermann *et al.*, 2021 ▸; Georgiev *et al.*, 2018 ▸; Pernpeintner *et al.*, 2017 ▸), inducing reversible modification of the layer thickness. Light-induced mesophase transitions studied by Wang *et al.* (2011 ▸) showed an irreversible transition from multilamellar vesicles to worm-like micelles. In liquid crystals, light-driven isomerization (Wuckert *et al.*, 2015 ▸), thermotropic phase transitions and structural reorientation (Bisoyi & Li, 2016 ▸; Yelamaggad *et al.*, 2012 ▸) are also observed.

In this study, we introduce a bilayer model system consisting of lipid mixtures of phospho­lipid 1,2-dipalmitoyl-phosphatidylcholine (DPPC) and synthetic photoswitchable lipid mimetics (Reise *et al.*, 2018 ▸) and use light to trigger phase transitions. Previous investigations by the authors into the structural response of mixed 95%/5% mol/mol Langmuir monolayers of DPPC and azo­benzene amphiphile moieties under illumination revealed bidirectional, repeatable and fully reversible changes of the layer thickness and surface pressure (Warias *et al.*, 2023 ▸). The switching stability and repeatability found in these monolayers make the azo­benzene amphiphiles ideal candidates for bilayer studies.

**Figure 1:**
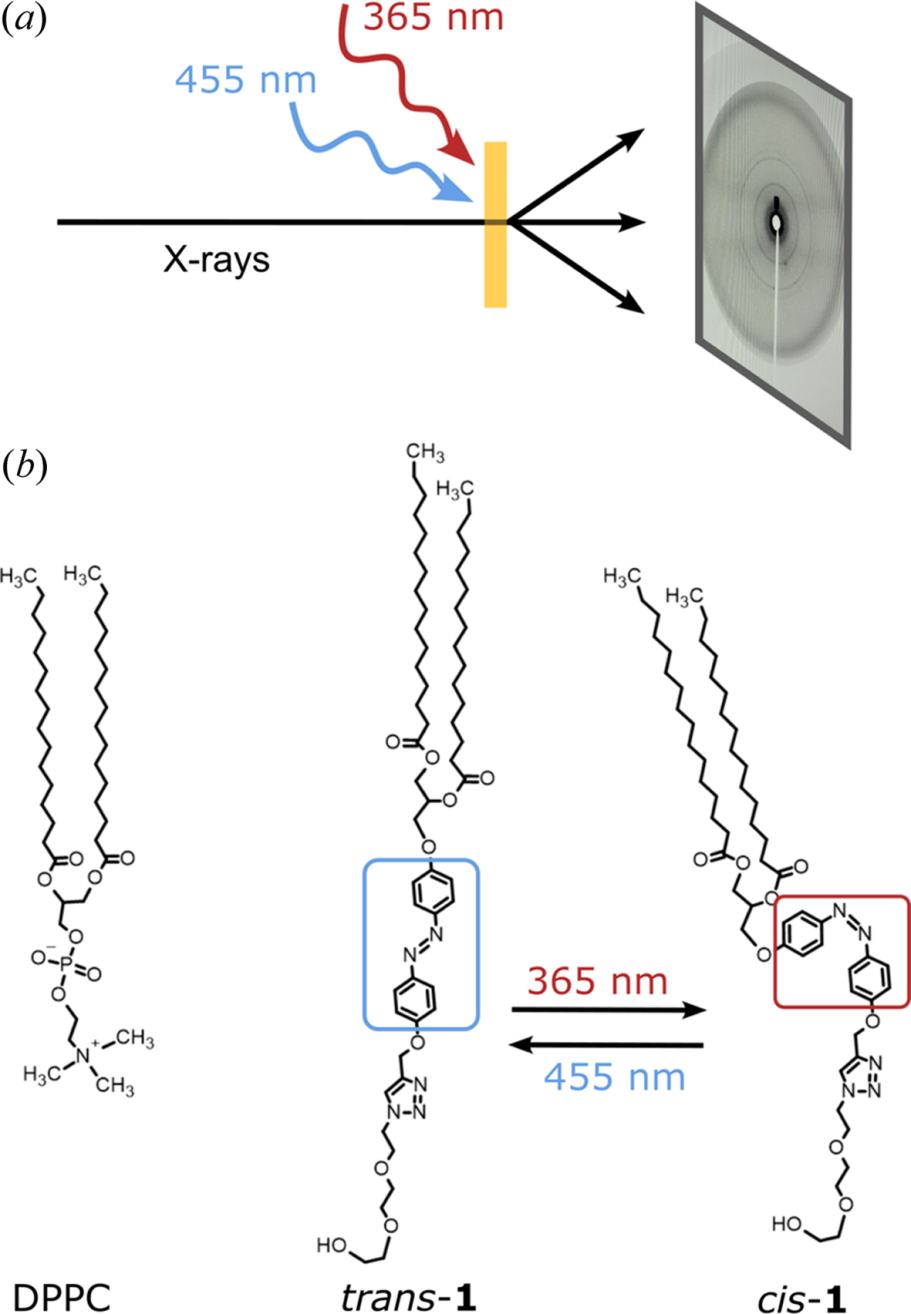
Schematic of (*a*) the transmission SAXS measurement setup and (*b*) the chemical structure of DPPC and both the *trans* and the *cis* isomers of azo­benzene amphiphile **1**.

The selected azo­benzene amphiphile is photosensitive and its N=N double bond switches between the straight *trans* and bent *cis* isomers on illumination with light of 365 and 455 nm wavelengths, respectively [see Fig. 1 ▸(*b*) for schematic representation]. In the employed synthetic azo­benzene amphiphiles, the azo­benzene moiety is attached on one side to a tri­ethyl­ene glycol linker forming the hydro­philic head group and on the other side to the hydro­phobic tail group consisting of di­acyl­glycerol esterified with two fatty acids containing 16 carbon atoms, similar to the DPPC tail.

In order to identify the lipid structures formed by mixtures of the azo­benzene amphiphiles and DPPC with proportions from 0 to 100% in water, small-angle X-ray scattering (SAXS) investigations were performed. SAXS is commonly employed to identify mesophases of lyotropic liquid crystals and to determine their scattering length profiles, sizes and shapes (Kikhney & Svergun, 2015 ▸; Kornmueller *et al.*, 2018 ▸; Hyde, 2001 ▸). In the following, we identify the mesophases and the light-induced phase transitions for the *trans* and *cis* isomers of the azo­benzene amphiphile, herein referred to as **1**. Furthermore, temperature-dependent SAXS measurements for selected proportions are analysed and complemented by differential scanning calorimetry (DSC) (McElhaney, 1982 ▸; Privalov & Plotnikov, 1989 ▸; Chiu & Prenner, 2011 ▸) measurements to determine the phase transition temperatures from gel to the liquid crystalline phase.

## 2. Experimental

### 2.1. Sample preparation

DPPC was purchased from Avanti Polar lipids (Alabaster, AL). The azo­benzene amphiphile **1** was synthesized in accordance with our previously published synthesis route (Reise *et al.*, 2018 ▸). The lipids were dissolved in chloro­form (Sigma–Aldrich) at a concentration of 10 m*M* and mixed at various proportions of DPPC and **1**. All following preparation steps were done for the stable *trans* form of **1**. These mixtures were dried to thin films with a BÜCHI Rotavapor R-300 at a bath temperature of 45°C and a pressure of 16 mbar for at least 1 h. These dried films were stored in the fridge at <10°C prior to the measurements. All samples were freshly prepared on the day of the measurements by adding warm Milli-Q water onto the film and then rotating and shaking the solution in a water bath above 45°C until a homogenous solution was formed. Larger lipid lumps were broken up using a vortex mixer and ultrasonic water bath. The hydrated samples were stored in the fridge at the beamline. Right before the measurements, the hydrated solutions were left for 1 h outside of the fridge to reach room temperature. Roti PreMix PBS salt (Carl Roth) (0.14 *M* NaCl, 2.7 m*M* KCl, 10 m*M* phosphate) was initially used as buffer to adjust the pH to 7.4 and later Milli-Q water was added. All measurements were performed no more than 24 h after the sample hydration.

### 2.2. Isomerization

A custom-made illumination device consisting of a row of three 365 nm LEDs [Nichia, NCSU033B(T)] and three 455 nm LEDs (Osram, LD CQ7P) was used to switch **1** from the *trans* to the *cis* isomer and back. Prior UV–Vis measurements on the azo­benzene mimetics revealed that 96% of the molecules switch to the *cis* and 82% to *trans* conformation (Warias *et al.*, 2023 ▸). Illumination and isomerization of the samples were carried out prior to both the DSC and the temperature-controlled SAXS measurements as the housing and sample holder, respectively, did not allow *in situ* illumination. The time following illumination between measurements was kept as short as possible to minimize back switching (see Section S1 of the supporting information). For the *in situ* SAXS measurements at room temperature, the illumination device was mounted above the capillary sample holder at a distance of about 10 cm. At the sample position, fluencies of 2.0 mW cm^−2^ for 365 and 455 nm were measured during the first beam time and 1.6 mW cm^−2^ during the second.

### 2.3. Differential scanning calorimetry

DSC measurements were carried out at a MicroCal Peaq-DSC (Malvern Panalytical, Northampton, MA) at P08 of PETRA III at DESY. After initial cleaning and reference measurements of pure and buffered Milli-Q water, sample solutions with 1 m*M* l^−1^ concentration were studied between 25 and 70°C at a heating rate of 12°C h^−1^ and a cooling rate of 60°C h^−1^. The heating and cooling scan was repeated three times. The data were recorded in high-feedback mode.

### 2.4. Small-angle X-ray scattering

All SAXS measurements were performed at BL2 of DELTA, Dortmund (Dargasz *et al.*, 2022 ▸) and at a concentration of 10 m*M* l^−1^. Two different sample setups were used to study temperature- and light-induced structural changes *in situ*. The schematic measurement setup is shown in Fig. 1 ▸(*a*). Temperature-dependent measurements were performed for lipid proportions of 0:100, 10:90 and 20:80 **1**:DPPC in the temperature range 17–65°C with a temperature-controlled cell (Linkam Scientific Instruments Ltd). In favour of *in situ* isomerization, a simple capillary sample holder was used for all other measurements at room temperature. The room temperature was monitored and varied between 18 and 27°C with a mode temperature of 25°C. For clarity, 25°C is given for all measurements between 18 and 27°C as the measured structures were identical in this range. The samples were filled in 1 and 2 mm diameter capillaries for the Linkam stage and simple sample holder, respectively. At DELTA, a photon energy of 12 keV, beam size of about 0.6 × 0.6 mm and a MAR345 2D image plate detector were used. The 2D detector images were processed with the software *FIT2D* (Hammersley *et al.*, 1995 ▸, 1996 ▸; Hammersley, 1997 ▸, 2016 ▸) including pixel mask application, transformation from real to reciprocal space, detector orientation correction and angular integration to receive the reduced 1D scattering pattern in *q*-space. Calibration of the detector distance and orientation were done with a standard silver behenate sample. The typical measurement time for the data collection was 180 s followed by a detector read out time of another 180 s.

### 2.5. Analysis software

A self-written Python script was used for background correction of the reduced 1D SAXS pattern and resaving the corrected pattern in the NeXus file format with the associated metadata (Wilkinson *et al.*, 2016 ▸). Following the metadata standards determined by DAPHNE4NFDI (Barty *et al.*, 2023 ▸), the newly generated NeXus file contains, in addition to the background-corrected pattern and the uncorrected signal, information on the background reduction as well as the detector, beamline and sample-specific metadata. The DSC data were also analysed with a self-written Python script and stored as simple ASCI files. Further information on the data and script accessibility are given in Data Availability[Sec sec5].

## 3. Results and discussion

### 3.1. Light-induced mesophase changes at room temperature

**Figure 2:**
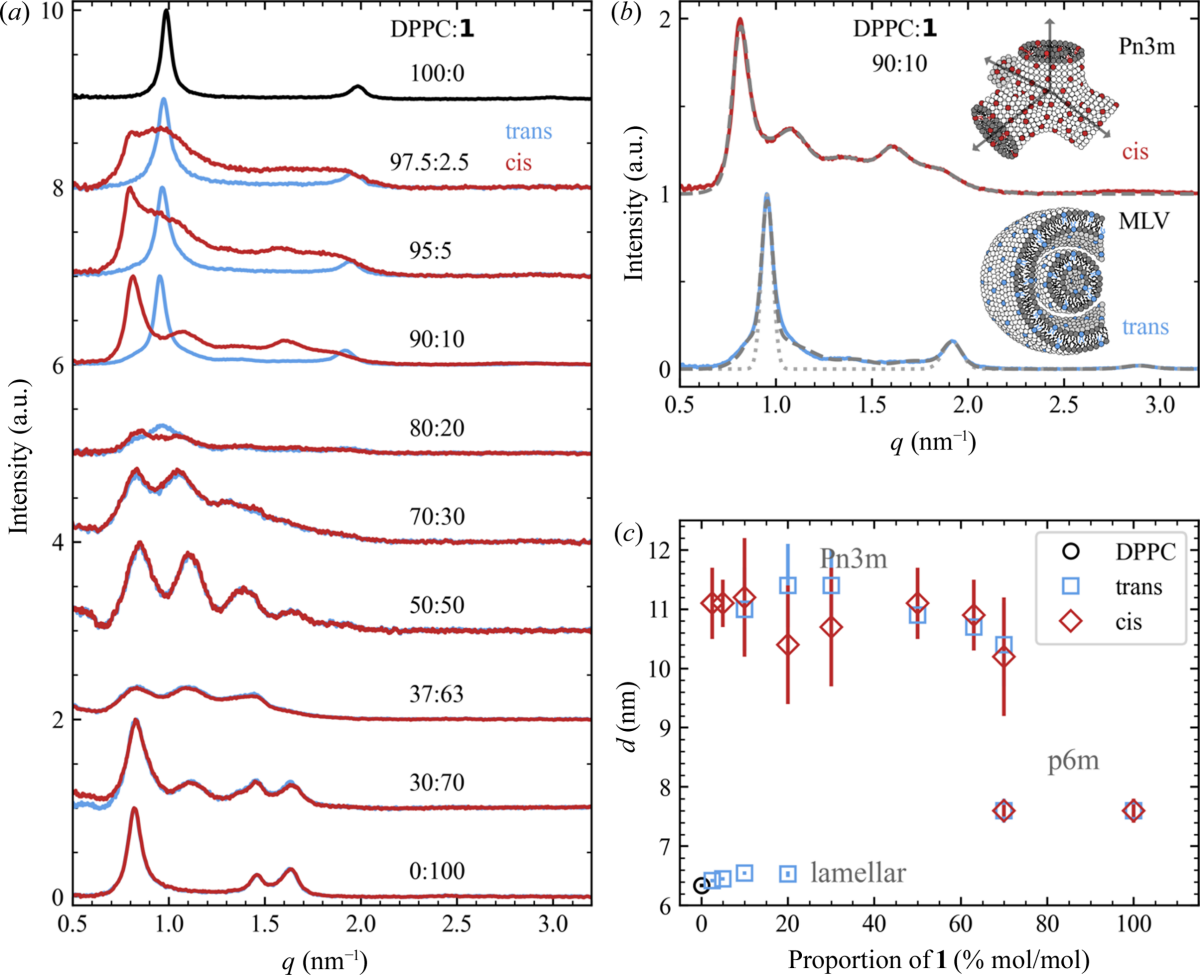
Scattering data of DPPC and **1** mixtures with varying proportions of **1** for both *trans* and *cis* isomers. (*a*) Pure DPPC (in black), mixed *trans*-**1** (in blue) and *cis*-**1** (in red) data. The curves with different proportions are offset vertically for clarity. (*b*) *Trans* and *cis* isomers of the mixture with 10% **1** and the structure fits (in grey) for the multilamellar vesicle (MLV) and *Pn*3*m* structure, respectively. For *trans*, the fits for the single lamellar (dotted), combined lamellar and *Pn*3*m* (dashed) phases are shown. The *d*-spacing values and mesophases derived from the fits are summarized in (*c*).

SAXS measurements on the hydrated samples for different proportions between 0 and 100% of DPPC and **1** in the *q*-range 0.5–3.2 nm^−1^ are shown in Fig. 2 ▸(*a*). A range of different mesophases are observed. At each concentration, we illuminated with 365 and 455 nm to measure the structures for the *cis* and *trans* states of **1**. Changes are observed between proportions of 2.5–20% of **1**.

For pure DPPC vesicles, the well studied multilamellar phase with a lamellar *d*-spacing of 6.34 ± 0.03 nm was found in agreement with previous studies (Soloviov *et al.*, 2012 ▸; Kornmueller *et al.*, 2018 ▸). Focusing on the *trans* isomer of **1**, a multilamellar vesicle (MLV) structure was also found for low proportions up to a molar percentage of 10% with an increase of the lamellar *d*-spacing up to 6.55 ± 0.03 nm. Increasing the proportion of **1** further, we observed a transformation to the cubic phase *Pn*3*m* for proportions between 20 and 70%, as shown in Fig. 2 ▸(*a*). At 70%, we found a coexistence of the previous cubic phase at low proportions and the hexagonal phase determined by fitting at 100%. Example raw detector images are presented in Fig. S1(*b*) of the supporting information, showing scattering from different mesophases.

Following *in situ* illumination of the mixed samples for 3–5 min with 365 nm, we could study the light-induced structural changes between the *trans* and *cis* isomer of **1**. Time-resolved measurements were not possible at DELTA, as the dynamics were faster than the counting and detector readout time. For proportions above 30% **1**, no light-induced change was observed. In contrast, we found a change in mesophase for proportions of **1** up to 20%. The lamellar phase with a lamellar *d*-spacing of 6.55 ± 0.03 nm for the *trans* isomer transformed to the bicontinuous cubic diamond *Pn*3*m* phase with a *d*-spacing of 11.2 ± 1.0 nm for the *cis* isomer is presented in Fig. 2 ▸(*b*) for the 10% proportion. This phase transformation was reversible by illumination with 455 nm light and was highly reproducible, as demonstrated for at least three illumination cycles (see Fig. S1). Based on the *Pn*3*m* phase identified for the *cis* isomer at 10 and 20% **1**, a coexistence of the lamellar and *Pn*3*m* phases was found for the corresponding *trans* isomer of **1** with *Pn*3*m* fractions of 59 ± 1% and 76 ± 1%, respectively. This coexistence was found for repetitive illuminations, showing a stable phase formation. By increasing the proportion of **1** to 30%, the *Pn*3*m* phase with *d*-spacings of 11.4 ± 0.6 and 10.7 ± 1.0 nm was found for both the *trans* and the *cis* isomers, respectively. At even higher proportions of 50 and 63%, not only the *Pn*3*m* phase but also the bicontinuous cubic *Im*3*m* structure give a reasonable fit, as described in Section S3 of the supporting information. The different mesophases revealed and *d*-spacing depending on the proportions between DPPC and **1** are shown in Fig. 2 ▸(*c*) and are summarized in Table 1 ▸.

The transformation to a cubic phase for a high content of **1** can be explained by an increase in the curvature of the monolayers, resulting in a rearrangement of the 3D structure. To quantify the curvature and the resulting self-assembled lipid geometry, the critical packing parameter (*CPP*) can be used to predict the phase, as introduced by Israelachvili *et al.* (1976 ▸) and Tanford (1973 ▸):
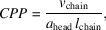
where *v*_chain_ and *l*_chain_ are the volume and length of the hydro­phobic chain, respectively, and *a*_head_ is the effective area of the hydro­philic headgroup. If *CPP* ≃ 1.0, the molecule can be considered to be cylindrical. A deviation of *CPP* from 1.0 indicates a positive or negative curvature which causes a cone-shape of the lipids and reformation into cubic, hexagonal or micellar structures, or their reversed correspondent. Flexible lipid bilayers are found for *CPP* values between 1.0 and 0.5, which can form both vesicles and cubic phases. When 0.3 < *CPP* < 0.5, the hexagonal structure is observed, and below 0.3 micelles are observed (Iakimov *et al.*, 2021 ▸; Charles Tanford, 1973 ▸; Nagarajan, 2002 ▸).

Theoretical values for the *CPP* factors of DPPC and **1**, in both the *trans* and the *cis* states, were estimated following the approach by Kobierski *et al.* (2022 ▸) and Oremusová *et al.* (2019 ▸). The detailed calculations and parameters are listed in Section S4 of the supporting information. In contrast to DPPC, **1** has *CPP* values of about 0.486 ± 0.002 and 0.440 ± 0.002 for the *trans* and *cis* isomers, respectively, suggesting a rod-like shape forming a hexagonal structure for pure **1**. Assuming the *CPP* values for the mixtures of DPPC and **1** can be calculated proportionally (König *et al.*, 2023 ▸), we observe a decrease in the *CPP* value with increasing concentration of **1** (see Table 1 ▸), which is in good agreement with the experimentally observed phases and the conversion from flexible bilayers to hexagonal structures at proportions of 70% **1**. Comparing the *CPP* values for *trans*-**1** and *cis*-**1**, the *cis* isomer is only slightly more curved, which could hint at favouring the cubic phase. Nevertheless, the *CPP* values for the *trans* and *cis* states are very close at low proportions and cannot be the only driving factor for the structural reorientation. Similar conclusions were drawn in recent molecular dynamic studies, where they revealed a lamellar-to-hexagonal transition on changes in temperature but with neglectable changes in the *CPP* value (Mochizuki, 2023 ▸).

### 3.2. Temperature-induced mesophase changes

**Figure 3:**
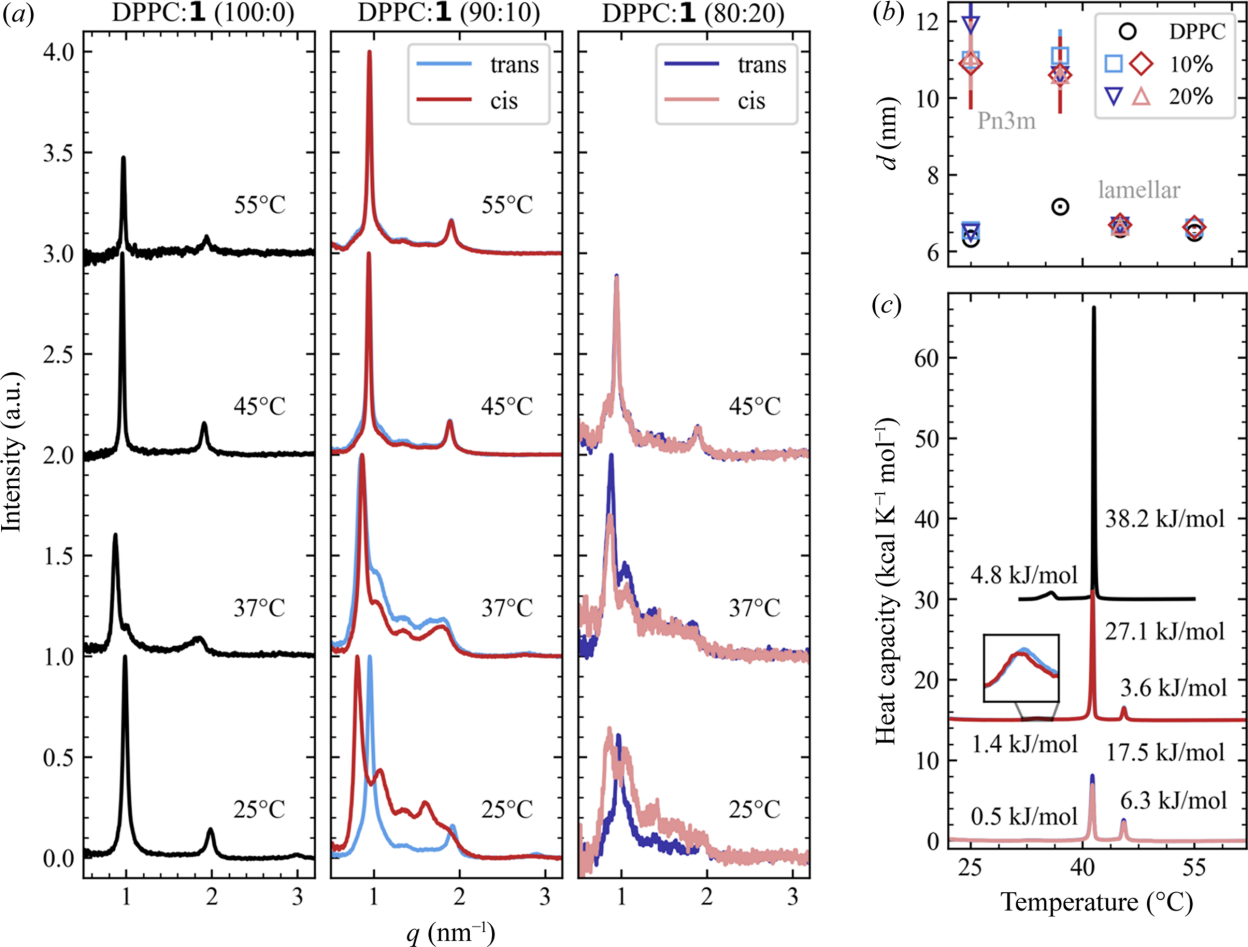
(*a*) SAXS data at different temperatures for mixed DPPC and **1** structures with proportion of 0, 10 and 20% **1**. The curves are shifted vertically and on the left the data from pure DPPC vesicles are shown. In the middle and right, respectively, the 10 and 20% mixtures of DPPC and **1** for both the *trans* (blue) and the *cis* states (red) are illustrated. The fitted *d*-spacing parameters and mesophases are summarized in (*b*) and complementary DSC data are shown in (*c*) for pure DPPC and mixtures of 10 and 20% **1** in the *trans* (blue) and *cis* states (red).

Samples were illuminated *ex situ* and later temperature-controlled measurements were performed using the Linkam stage. Two capillaries were filled from the same stock solution and both were illuminated with 365 nm light for 5 min for *tans*–*cis* isomerization. Afterwards, one capillary was illuminated with 455 nm to transform back to the *trans* state. The *cis* isomer was measured first, followed by the thermally stable *trans* form. The temperature was increased from room temperature (25°C) in steps up to 55°C at a heating rate of 10°C min^−1^. After reaching the desired temperature, a 10 min waiting time was introduced before starting each SAXS measurement. These temperature-dependent measurements were carried out for mixtures of 10 and 20% **1** and pure DPPC, and the data are presented in Fig. 3 ▸(*a*). The observed Bragg peak shifts and calculated lamellar *d*-spacing for pure DPPC are in good agreement with other studies of multilamellar DPPC vesicles (Almeida *et al.*, 2018 ▸), except for the rippled phase expected at 37°C, which was absent. The reason for not observing the rippled phase for pure DPPC could be due to a temperature offset between the set temperature and the sample temperature (typically between 0.5 and 1°C) or due to not waiting long enough to allow the formation of the rippled phase.

Comparing the heating series for the *trans* and *cis* isomers of 10% **1**, the mesophases are identical for both isomers at temperatures higher than 37°C [see Fig. 3 ▸(*a*)]. A clear difference between the lamellar (*trans*) and *Pn*3*m* (*cis*) phase was only observed at room temperature. The fitted structures and *d*-spacings match the *in situ* measurements in Fig. 2 ▸(*a*). At 37°C, we found a *Pn*3*m* structure for both isomers, *trans* and *cis*, with a slightly decreased *d*-spacing of 11.1 ± 0.7 and 10.6 ± 1.0 nm [see Table 1 ▸ and Fig. 3 ▸(*b*)]. When the temperature was increased above the main phase transition to 45 and 55°C, a transition back to the lamellar phase occurred with lamellar *d*-spacings of 6.64 ± 0.03 and 6.62 ± 0.03 nm, respectively, for the *trans* isomer. This back transition could be attributed to the higher flexibility of the tails in the liquid crystalline phase, causing the tail length to appear shorter, which results in an increased *CPP* value.

Analogue transitions were observed for 20% **1** in *trans* and *cis* [see Figs. 3 ▸(*a*) and 3 ▸(*b*)]. In contrast to the *trans* isomer, the *cis* isomer only shows two phase transitions as the *Pn*3*m* structure is already present at room temperature. Complementary DSC measurements showed three phase transitions for 10 and 20% **1** [see Fig. 3 ▸(*c*)], supporting the phase transitions observed in the SAXS data. For 20%, the pre-transition peak at 33.1 ± 0.3°C, belonging to the DPPC molecules, was hardly observable with an enthalpy of only 0.5 ± 0.1 kJ mol^−1^. A decrease in the enthalpy is typically found for mixed systems with increasing proportions of the second compound, as shown by Almeida *et al.* (2018 ▸) for mixed DPPC and cholesterol vesicles. Generally, a reduction in the enthalpy for each phase transition, a shift towards lower temperatures and an emerging additional phase transition peak at around 45.5°C were found on increasing the proportion of **1** [see Fig. 3 ▸(*c*)]. Meanwhile, the difference between the *trans* and *cis* phase transition temperatures is negligibly small and within the measurement error for the phase transitions at 41.3 ± 0.3 and 45.5 ± 0.3°C. The shift of 0.3°C between the pre-phase transition temperatures 33.7 ± 0.3°C and 44.0 ± 0.3°C for 10% **1** in *trans* and *cis*, respectively, is close to the measurement accuracy.

## 4. Conclusions

In summary, from the SAXS investigation on mixed DPPC and the photoswitchable azo­benzene amphiphile **1** in aqueous solutions, we could confirm reversible light-induced switching between mesophases. Photoswitching was observed for concentrations up to 20% of the photosensitive molecule **1** and for temperatures from 18 to 27°C which correspond to the lipid gel phase. The mesophase could be switched reversibly, repeatedly and reproducibly between its lamellar structure in the *trans* state and its cubic *Pn*3*m* structure in the *cis* state. For proportions higher than 20% **1**, the cubic *Pn*3*m* and at even higher proportions such as 70% a hexagonal structure was favoured for both the *trans* and the *cis* states equally, showing the sensitivity of the lipid structures to the lipid composition.

DSC measurements identified the phase transition temperatures. Complementary temperature-dependent SAXS measurements for mixtures containing 10% **1** in the *trans* state showed a mesophase transition from lamellar to cubic *Pn*3*m* at the 34.0 ± 0.3°C phase transition and a mesophase transition from *Pn*3*m* back to lamellar at the main phase transition at 41.4 ± 0.3°C. Interestingly, no mesophase transition was observed at 33.7 ± 0.3°C for the *cis* state, which is already present in the *Pn*3*m* phase at room temperature, but similar to the *trans* state displays a transition to lamellar at 41.3 ± 0.3°C. At temperatures above 34°C, no photoswitching was observed, as here both the *trans* and the *cis* forms are present in the same mesophase.

These findings indicate a useful temperature and concentration range for photoswitching applications. The first steps to create model systems for the study of membrane protein response to mesophase changes due to external stimuli without altering the environment are shown. Further, such systems may have potential as a basis for drug delivery applications.

## 5. Data availability

Raw data, fit parameters and analysis scripts are available under: https://doi.org/10.57892/100-49 International Generic Sample Numbers (IGSNs): DPPC:**1** 100:0 (https://doi.org/10.60578/gucn-en2h); DPPC:**1** 97.5:2.5 (https://doi.org/10.60578/kw2n-ouv5); DPPC:**1** 95:5 (https://doi.org/10.60578/dg1l-5gebr); DPPC:**1** 90:10 (https://doi.org/10.60578/reqz-rk9v); DPPC:**1** 80:20 (https://doi.org/10.60578/r2j8-grzb); DPPC:**1** 70:30 (https://doi.org/10.60578/l7hw-iao2); DPPC:**1** 50:50 (https://doi.org/10.60578/p1fg-n3tw); DPPC:**1** 37:63 (https://doi.org/10.60578/g7n3-wkj3); DPPC:**1** 30:70 (https://doi.org/10.60578/q7j3-sid5); DPPC:**1** 0:100 (https://doi.org/10.60578/xke5-nz1u).

## 6. Related literature

The following references are cited in the supporting information: Ishiwatari *et al.* (2024 ▸); Khalil & Zarari (2014 ▸); SzymaŃski *et al.* (2013 ▸); Tanford, C. (1972 ▸).

## Supplementary Material

Supporting figures, SAXS procedures and CPP calculations. DOI: 10.1107/S2052252524004032/zf5025sup1.pdf

Link to the raw data, fit results and python scripts.: https://opendata.uni-kiel.de/receive/fdr_mods_00000049?accesskey=PUxyFPKUj2B1aXRh82nPtWW4Y2E7hgwW

## Figures and Tables

**Table:** The error for all estimated *CPP* values is ±0.002.

*T* (°C)	DPPC:**1**	Mesophase	*d* (nm)	*CPP*
25	100:0	Lamellar	6.34 ± 0.03	0.6†
37	100:0	Lamellar	7.17 ± 0.05	
45	100:0	Lamellar	6.58 ± 0.01	
55	100:0	Lamellar	6.49 ± 0.01	

**Table:** 

		*Trans*			*Cis*		
*T* (°C)	DPPC:**1**	Mesophase	*d* (nm)	*CPP*	Mesophase	*d* (nm)	*CPP*
25	97.5:2.5	Lamellar	6.42 ± 0.03	0.597	*Pn*3*m*	11.1 ± 0.6	0.596
25	95:5	Lamellar	6.45 ± 0.03	0.594	*Pn*3*m*	11.1 ± 0.4	0.592
25	90:10	Lamellar, *Pn*3*m*	6.55 ± 0.03, 11.0 ± 0.7	0.589	*Pn*3*m*	11.2 ± 1.0	0.584
25	80:20	Lamellar, *Pn*3*m*	6.53 ± 0.05, 11.4 ± 0.7	0.577	*Pn*3*m*	10.4 ± 1.0	0.568
25	70:30	*Pn*3*m*	11.4 ± 0.6	0.566	*Pn*3*m*	10.7 ± 1.0	0.552
25	50:50	*Pn*3*m*	10.9 ± 0.4	0.543	*Pn*3*m*	11.1 ± 0.6	0.52
25	37:63	*Pn*3*m*	10.7 ± 0.4	0.528	*Pn*3*m*	10.9 ± 0.6	0.499
25	30:70	*Pn*3*m*, *p*6*m*	10.4 ± 0.7, 7.6 ± 0.2	0.520	*Pn*3*mp*6*m*	10.2 ± 1.0 7.6 ± 0.2	0.488
25	0:100	*p*6*m*	7.6 ± 0.2	0.486	*p*6*m*	7.6 ± 0.2	0.440

25	90:10	Lamellar, *Pn*3*m*	6.55 ± 0.03, 11.0 ± 0.7		*Pn*3*m*	10.9 ± 1.2	
37	90:10	*Pn*3*m*	11.1 ± 0.7		*Pn*3*m*	10.6 ± 1.0	
45	90:10	Lamellar	6.64 ± 0.03		Lamellar	6.69 ± 0.03	
55	90:10	Lamellar	6.62 ± 0.03		Lamellar	6.63 ± 0.03	
25	80:20	Lamellar, *Pn*3*m*	6.49 ± 0.01, 11.9 ± 0.8		*Pn*3*m*	11.1 ± 0.9	
37	80:20	*Pn*3*m*	10.6 ± 0.4		*Pn*3*m*	10.6 ± 0.4	
45	80:20	Lamellar	6.67 ± 0.01		Lamellar	6.64 ± 0.01	

† Reference value for DPPC was taken from Kobierski *et al.* (2022 ▸).
