# Supplementary material for: Photoinduced bidirectional mesophase transition in vesicles containing azo­benzene amphiphiles
Source: IUCrJ. 2024 May 28;11(Pt 4):486–93. doi: 10.1107/S2052252524004032 (PMC11220873; doi:10.1107/S2052252524004032)
Supplement: Supplementary file 1 [file m-11-00486-sup1.pdf]

IUCrJ

Volume 11 (2024)

Supporting information for article:

Photoinduced bidirectional mesophase transition in vesicles containing azobenzene amphiphiles

Svenja C. Hövelmann, Ella Dieball, Jule Kuhn, Michelle Dargasz, Rajendra P. Giri, Franziska Reise, Michael Paulus, Thisbe K. Lindhorst and Bridget M. Murphy

S1. *Cis* stability tests

During the DSC and SAXS measurements possible thermal back isomerisation was minimised by keeping the time between the measurement and illumination as short as possible. For the SAXS measurements, we made a prior test on the *cis* stability of the mixture with 20% of **1** at 37°C. For that, we illuminate the sample for three illumination cycles (UV-blue-UV-blue) and kept the sample in the temperature controlled Linkam stage at 37°C. The LED Box was positioned in front of the sample holder window (5 mm diameter) for the X-ray beam for about 5 minutes. After removing the LED Box and searching the hutch (below 1 minute), the SAXS pattern was measured immediately. This procedure was repeated at 45°C for one illumination cycle. No difference in the SAXS signals were observed after the illumination. For the 10% of **1** SAXS data, the sample was illuminated for 5 minutes before starting the temperature run 2 minutes later. The three-step temperature run from 37°C to 55°C took about 90 minutes. In the case of DSC measurements, the time between illumination and the start of the measurement was about 90 minutes and the measuring time was about 105 minutes.

S2. Multiple illumination cycles and raw detector images

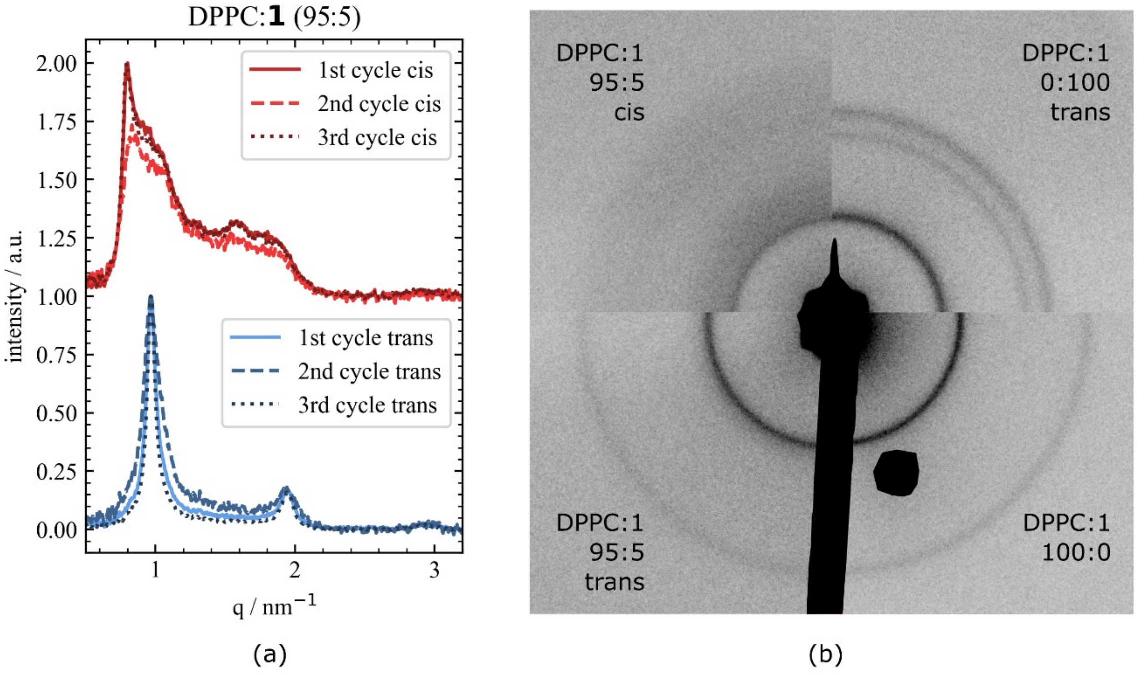

Figure S1 a) SAXS data obtained from the DPPC and 5 % **1** mixtures for its *trans* and *cis* isomers after iterative illumination of 365 nm and 455 nm, respectively, to switch three times between *cis*-**1** and *trans*-**1**. b) Detector images from 95:5 DPPC:**1** *cis*-state (top left) and *trans*-state (bottom left), 100% *trans*-**1** (top right) and 100% DPPC (bottom right).

S3. Fitting different mesophases

All SAXS data was fitted using a two-step approach to determine the mesophase structure. In the first step the fit model consisted of multiple Gaussian-functions with variable amplitude $a_{1,2,\ldots,\mathrm{n}}$, width $\sigma_{1,2,\ldots,\mathrm{n}}$ and d-spacing d.

$$f(q) = a_1\, e^{-\frac{(q-p_1)^2}{2\sigma_1^2}} + \cdots + a_\mathrm{n}\, e^{-\frac{(q-p_\mathrm{n})^2}{2\sigma_\mathrm{n}^2}}$$

The positions $p_{1,2,\ldots,\mathrm{n}}$ of the Gaussian peaks were calculated using the d-spacing parameter d for the lamellar, hexagonal p6m and the bicontinuous cubic phases Pn3m, Im3m, Ia3d, Fm3m, Pm3n and Fd3m as for example for the lamellar phase: $p_\mathrm{n} = \frac{2\pi}{d} n$, or for the hexagonal p6m phase: $p_1 = \frac{2\pi}{d}\sqrt{1}$, $p_2 = \frac{2\pi}{d}\sqrt{3},\ p_3 = \frac{2\pi}{d}\sqrt{4}$, ... The space groups are taken from (Hyde, 2001).

A visual check was introduced to choose the best match. In most cases only one phase was in good agreement with the measured data. This phase was then selected for the second step, small deviations $\delta_{1,2,\ldots,\mathrm{n}}$ of the peak positions for each peak in the scattering data was allowed.

$$f(q) = a_1\, e^{-\frac{(q-(p_1+\delta_1))^2}{2\sigma_1^2}} + \cdots + a_\mathrm{n}\, e^{-\frac{(q-(p_\mathrm{n}+\delta_\mathrm{n}))^2}{2\sigma_\mathrm{n}^2}}$$

For all positions $p_\mathrm{n} + \delta_\mathrm{n}$, the d-spacing parameters were recalculated and averaged. The error for the d-spacing value was then calculated from the deviations. The fit results are accessible with the raw data (see Data Availability).

For some ratios of **1**, such as for 50% and 63 %, either the Pn3m or Im3m phase give a good fit to the data as shown in Fig. S2. For lower ratios such as 30 % of **1**, the Pn3m fit shows better accordance than the Im3m structure (see Fig. S2).

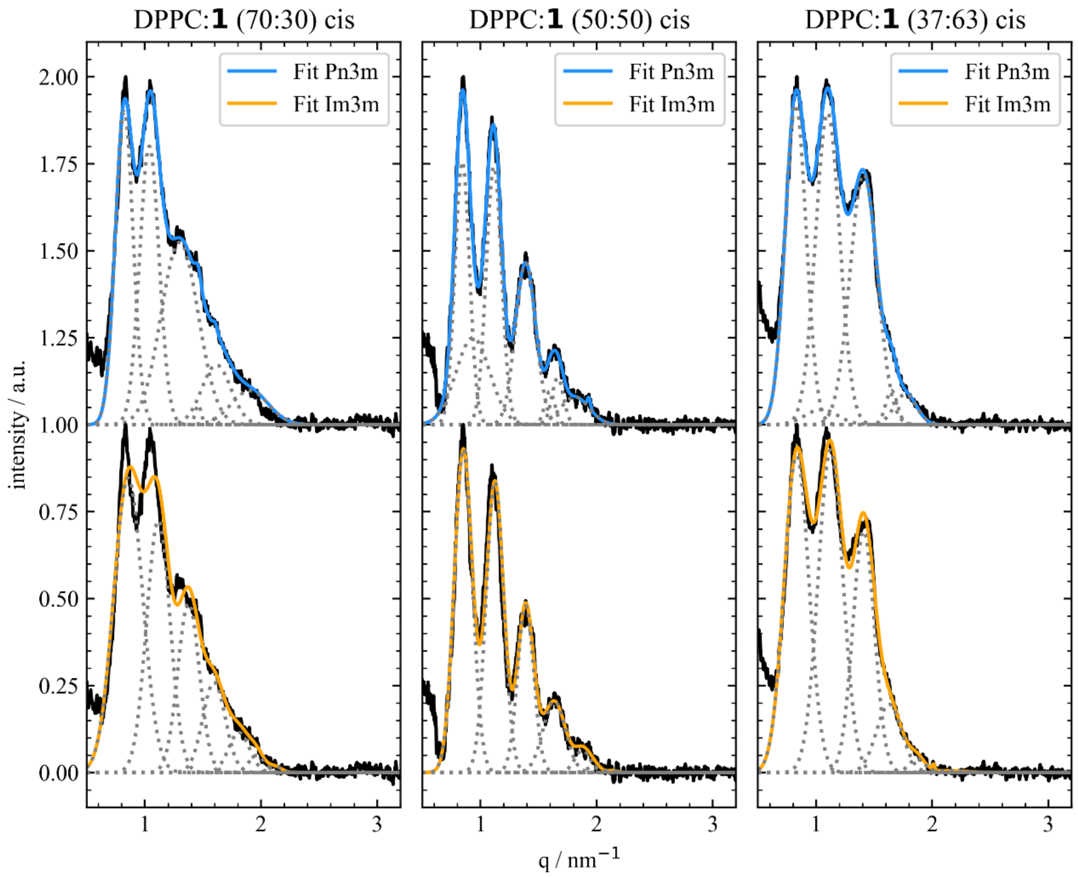

Figure S2 SAXS data obtained from the mixed DPPC with 30 % (left), 50 % (middle) and 63 % (right), respectively, *cis*-**1** shown twice with the fits for the Pn3m structure (top) and Im3m structure (bottom).

S4. Calculation of critical packing parameter

For the estimation of the maximum alkyl chain length l_{chain} and volume v_{chain} the following equations were used: (Tanford, 1972)

$$l_{\mathrm{chain}} \leq l_{\max} = 1.5 + 1.265 * n \; [\text{Å}]$$

$$v_{\mathrm{chain}} = 54.3 + 27.05 * 2n \; [\text{Å}^3]$$

where n represents the number of carbon atoms in one acyl chain. For both, DPPC and **1**, the acyl chain consists of 16 carbon atoms, resulting in $l_{\mathrm{chain}} = 21.74$ Å and $v_{\mathrm{chain}} = 919.9$ Å^3. As the acyl chains are not fully stretched in reality, values from a molecular dynamic simulation by (Kobierski *et al.*, 2022) at 20°C are used to get a more accurate estimation: $l_{\mathrm{chain}} = 19.22$ Å and $v_{\mathrm{chain}} = 722.57$ Å^3. They calculated the hydrophobic head area to 62.71 Å^2 and a resulting critical packing parameter CPP value of 0.6 for pure DPPC.

Calculating the area of hydrophilic head group is not trivial and different experimental and theoretical approaches report different values. New molecular models are used also in combination with machine learning to get a better understanding and more accurate values. (Kobierski *et al.*, 2022; Ishiwatari *et al.*, 2024; Khalil & Zarari, 2014) In this work, molecular modulations of our specific molecule **1** were not possible and therefore only an approximation of the hydrophilic head group area a_{head} is presented. To estimate a_{head} for the **1** isomer, the observed difference in area per molecule for the LE-LC phase transitions determined in Langmuir monolayer studies of mixed 90:10 DPPC:**1** at 21°C were taken. A difference of (14.7 ± 0.2) Å^2 and (22.8 ± 0.2) Å^2 between the LE-LC phase transition of DPPC and **1** *trans* and *cis*, respectively, were observed. Adding these values to the theoretical head group area of 62.71 Å^2 for DPPC the following values could be derived:

$$a_{\mathrm{head},\mathbf{1},\mathrm{trans}} = (77.4 \pm 0.2)\ \text{Å}^2$$

$$a_{\mathrm{head},\mathbf{1},\mathrm{cis}} = (85.5 \pm 0.2)\ \text{Å}^2.$$

Under the assumption, that the hydrophobic tail packing is independent of the polar head group, the chain length and volume modelled for DPPC can be assumed for **1** as well. This results in CPP values of 0.486 ± 0.002 and 0.440 ± 0.002 for the *trans*-**1** and *cis*-**1** isomer, respectively. The decrease of the CPP value upon cis isomerisation agrees with other studies on photoisomerisation molecules (Szymański *et al.*, 2013; Wang *et al.*, 2011).

References

Hyde, S. T. (2001). *Handbook of Applied Surface and Colloid Chemistry*, edited by Krister Holmberg, pp. 299–327, John Wiley & Sons, Ltd.

Ishiwatari, Y., Yokoyama, T., Kojima, T., Banno, T. & Arai, N. (2024). *Mol. Syst. Des. Eng.* **9**, 20–28, doi:10.1039/D3ME00151B.

Khalil, R. A. & Zarari, A. A. (2014). *Applied Surface Science*. **318**, 85–89, doi:10.1016/j.apsusc.2014.01.046.

Kobierski, J., Wnętrzak, A., Chachaj-Brekiesz, A. & Dynarowicz-Latka, P. (2022). *Colloids and surfaces. B, Biointerfaces*. **211**, 112298, doi:10.1016/j.colsurfb.2021.112298.

Szymański, W., Yilmaz, D., Koçer, A. & Feringa, B. L. (2013). *Accounts of chemical research*. **46**, 2910–2923, doi:10.1021/ar4000357.

Tanford, C. (1972). *The Journal of Physical Chemistry*. **76**, 3020–3024, doi:10.1021/j100665a018.

Wang, D., Dong, R., Long, P. & Hao, J. (2011). *Soft Matter*. **7**, 10713, doi:10.1039/C1SM05949A.
